# A Case Report of Pancreatic Oligometastasis of Uterine Leiomyosarcoma

**DOI:** 10.1155/crom/1838527

**Published:** 2026-04-15

**Authors:** Anne Cambrelin, Sydney Oesch, Brian Orr, Chelsea Wooster, David Lewin, Justin Harold

**Affiliations:** ^1^ Medical University of South Carolina, Charleston, South Carolina, USA, musc.edu; ^2^ Division of Gynecologic Oncology, Department of Obstetrics and Gynecology, Medical University of South Carolina, Charleston, South Carolina, USA, musc.edu; ^3^ Department of Pathology and Laboratory Medicine, Medical University of South Carolina, Charleston, South Carolina, USA, musc.edu

**Keywords:** oligometastatic disease, pancreatic metastasis, uterine leiomyosarcoma

## Abstract

**Background:**

Uterine leiomyosarcoma (uLMS) is a rare, aggressive uterine malignancy with poor prognosis due to its early hematogenous spread, typically to the lungs and liver, and high recurrence rate. Pancreatic metastases are exceptionally rare, and isolated involvement of the pancreas is even more uncommon.

**Case:**

An 81‐year‐old patient was admitted after an unwitnessed fall and new neurologic symptoms, later diagnosed as a cerebellar stroke. Abdominopelvic imaging for urinary retention revealed a 9.9 × 15.1 × 18.8 − cm heterogeneous uterine mass and a 1.9‐cm hypoenhancing lesion in the pancreatic head. Pelvic MRI findings suggested uLMS. Endoscopic ultrasound–guided biopsy confirmed the pancreatic lesion as metastatic leiomyosarcoma. No other sites of metastatic disease were found. Due to her extensive comorbidities and limited performance status, the patient ultimately elected for hospice care after being thoroughly counseled on her treatment options and multidisciplinary consultation.

**Conclusion:**

This case illustrates a rare instance of isolated pancreatic metastasis from primary uLMS, a presentation that can mimic primary pancreatic cancer. It emphasizes the need for a broad differential diagnosis, histologic confirmation, and individualized treatment planning in medically complex patients.

**Highlights**

• Isolated pancreatic metastasis of uterine leiomyosarcoma (uLMS) at initial diagnosis is exceptionally rare and expands the recognized metastatic spectrum of uLMS.
• This presentation mimicked a synchronous primary pancreatic malignancy, highlighting diagnostic ambiguity and the need for histologic confirmation.
• Endoscopic ultrasound (EUS)–guided biopsy enabled definitive diagnosis when percutaneous biopsy was technically not feasible.
• Multidisciplinary evaluation, diagnostic accuracy, and patient‐centered decision‐making were essential given the rarity of this cancer type, metastatic pattern, and the patient’s medical complexity.

## 1. Background

uLMS accounts for only 2%–5% of all uterine malignancies [1] but contributes disproportionately to morbidity and mortality. Treatment of uLMS is challenging due to its aggressive biology and limited responsiveness to conventional therapies. The disease is marked by early hematogenous dissemination and high rates of recurrence. The 5‐year survival rate is only 10%–15% in patients presenting with metastatic disease [2].

Initial symptoms are often nonspecific and may resemble benign uterine conditions such as fibroids, contributing to delayed diagnosis. At the time of presentation, approximately 30%–35% of patients already have distant metastases [3]. The lungs are the most common site of metastasis, followed by the brain, skin, soft tissues, and bone [4].

Metastasis to the pancreas is exceedingly rare. Reports of pancreatic metastases from uLMS have been reported in the setting of upfront widely metastatic disease or in recurrent disease. Metastasis of uLMS to the pancreas is exceedingly rare and has been described almost exclusively in the setting of widely disseminated or recurrent disease. Isolated pancreatic involvement without concurrent metastases to more typical sites such as the lungs or liver has not been reported at initial presentation, a pattern that may mimic a second primary malignancy and necessitates a careful diagnostic approach. To our knowledge, this represents the first reported case of isolated pancreatic metastasis identified at the time of initial uLMS diagnosis.

## 2. Case Presentation

An 81‐year‐old patient with a medical history of atrial fibrillation, hypertension, hyperlipidemia, and multiple deep vein thromboses was hospitalized following an unwitnessed fall in the setting of a recent ischemic cerebellar stroke. During her admission, she developed urinary retention and underwent abdominopelvic imaging that revealed a large pelvic mass. She denied abdominal pain, vaginal bleeding, or weight loss. Laboratory studies revealed a normocytic anemia (hemoglobin 8.5 g/dL, mean corpuscular volume [MCV] 81.2 fL) and hypoalbuminemia of 2.7 g/dL. Kidney and liver function were within normal limits. Her tumor markers were notable for an elevated Cancer Antigen 125 (CA‐125) of 61 U/mL and a Cancer Antigen 19‐9 (CA 19‐9) and carcinoembryonic antigen (CEA) within the normal range.

Computed tomography (CT) imaging showed a 9.9 × 15.1 × 18.8 − cm heterogeneous pelvic mass and a 1.9‐cm hypoenhancing lesion in the pancreatic head without biliary or pancreatic ductal dilation. No other lesions were identified on chest, abdominal, or pelvic imaging. As part of her diagnostic evaluation, the patient also underwent colonoscopy, which identified two colonic polyps that were excised and demonstrated tubular adenoma on pathology without malignant transformation. These findings excluded a synchronous colorectal malignancy as the source of the pancreatic lesion.

**Figure 1:**
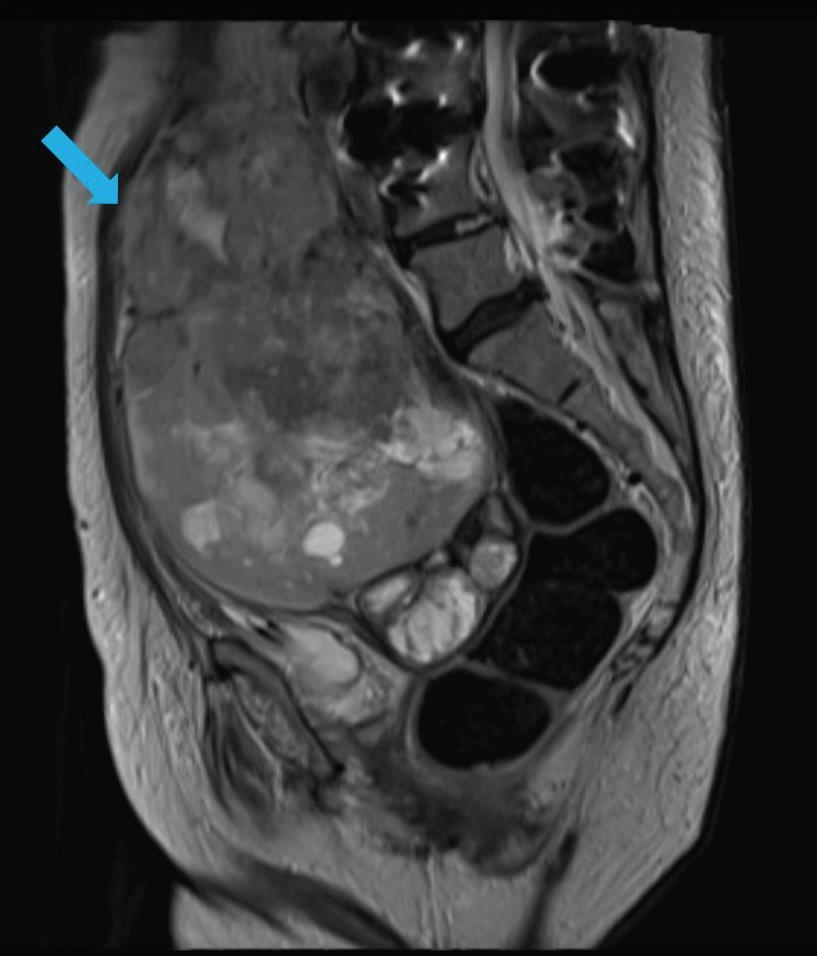
Sagittal T2‐weighted MRI demonstrating a large, heterogeneous uterine mass measuring 9.9 × 15.1 × 18.8 cm (blue arrow) with areas of necrosis and hemorrhage, consistent with primary uterine leiomyosarcoma.

**Figure 2:**
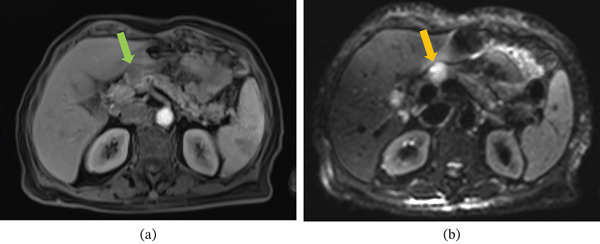
(a) Axial T1‐weighted MRI image demonstrating a 1.9‐cm hypoenhancing lesion in the pancreatic head (green arrow), consistent with a metastatic focus from uterine leiomyosarcoma. (b) Axial diffusion‐weighted MRI (DWI) image demonstrating restricted diffusion in the pancreatic head lesion (yellow arrow), consistent with a metastatic focus from uterine leiomyosarcoma.

MRI of the abdomen and pelvis demonstrated a large heterogeneous uterine mass with areas of central necrosis and hemorrhage, radiographically concerning for leiomyosarcoma (Figures 1 and 2).

Although uLMS is a histologic diagnosis, certain radiographic findings can raise suspicion for malignancy and prompt further evaluation. In this case, no endometrial or uterine biopsy was performed during hospitalization due to the patient′s significant comorbidities and clinical frailty, as well as the limited diagnostic sensitivity of endometrial sampling for uLMS, which yields a diagnosis in only approximately half of cases [5]. Furthermore, given the imaging findings, the patient was clinically staged as either having advanced uLMS with metastatic disease or localized uLMS with a primary pancreatic lesion. Thus, uterine tissue confirmation was not anticipated to alter immediate management decisions.

At presentation, two primary diagnostic pathways were considered: (1) metastatic uLMS, which would necessitate consideration of systemic therapy or highly morbid surgical interventions, or (2) localized uterine malignancy with a pancreatic head lesion of independent etiology. Characterization of the pancreatic lesion became the critical determinant of management. If the pancreatic lesion represented metastatic uLMS, treatment options would be limited to systemic therapy or aggressive resection, both of which carried prohibitive risk in this patient. Alternatively, if the pancreatic lesion represented a primary pancreatic malignancy, prognosis and treatment strategy would need to be established in conjunction with surgical and medical oncology prior to any gynecologic intervention. Finally, if the pancreatic lesion were benign, hysterectomy could be considered for definitive management of presumed uterine malignancy.

Percutaneous image‐guided biopsy was initially evaluated in consultation with interventional radiology (IR); however, no safe biopsy window was identified due to the lesion′s small size, central location within the pancreatic head, and proximity to surrounding vascular structures. Consequently, the case was discussed with interventional gastroenterology, and EUS‐guided biopsy was selected as the safest and most diagnostically appropriate approach.

**Figure 3:**
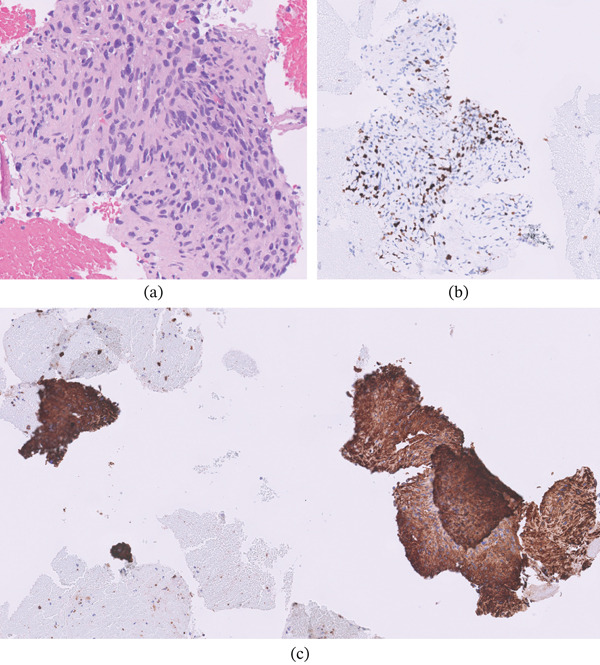
(a) Hematoxylin and eosin–stained tissue demonstrates disorganized intersecting fascicles with marked cytologic atypia. (b) Positive immunohistochemical staining with SMA (smooth muscle actin) supports a diagnosis of metastatic conventional (spindle cell) leiomyosarcoma in the clinical setting of a uterine mass. Negative immunohistochemical stains for cytokeratin, CD117, DOG‐1, and synaptophysin render a diagnosis of carcinoma, gastrointestinal stromal tumor, or neuroendocrine tumor unlikely. (c) A pathologic diagnosis of conventional leiomyosarcoma requires two of the following criteria: marked cytologic atypia, tumor cell necrosis, or ≥ 4 mitoses/mm2 (equating to ≥ 10 mitoses/10 HPF). Ki‐67 immunostaining of this biopsy demonstrates positivity in 30% of tumor cells, exceeding 10 mitoses per 10 HPF.

Pathologic evaluation revealed spindle cell neoplasm with smooth muscle differentiation consistent with metastatic leiomyosarcoma (Figure 3). Histologic analysis demonstrated marked cytologic atypia and a high mitotic index (≥ 10 mitoses per 10 high‐power fields), meeting two of the three established diagnostic criteria for leiomyosarcoma. Immunohistochemical staining was positive for smooth muscle actin (SMA) and desmin and negative for cytokeratin, CD117, DOG‐1, and synaptophysin, excluding epithelial, gastrointestinal stromal, and neuroendocrine neoplasms [6, 7]. Taken together with the MRI characteristics of the uterine mass, these findings supported a diagnosis of uLMS with isolated pancreatic metastasis.

The case was reviewed by gynecologic oncology, medical oncology, interventional gastroenterology, radiation oncology, and palliative care. Given her recent cerebellar stroke, paroxysmal atrial flutter, chronic anemia, and extensive venous thrombosis, the patient was deemed a poor candidate for radical surgical management (hysterectomy with concurrent pancreaticoduodenectomy). The risks and expected toxicities of systemic therapy—including doxorubicin monotherapy and in combination with trabectedin—were reviewed with the patient. After multidisciplinary discussion with gynecologic oncology, radiation oncology, and palliative care, the patient elected to pursue comfort‐focused hospice care.

## 3. Discussion

This case highlights a rare presentation of oligometastatic uLMS with isolated involvement of the pancreas. Pancreatic metastases from solid tumors are uncommon and are typically seen in the context of widely disseminated disease, most frequently arising from renal cell carcinoma, lung cancer, or melanoma [8]. Metastatic spread to the pancreas from gynecologic malignancies is particularly rare. The pancreas′ rich capillary network and dual blood supply might suggest vulnerability to hematogenous spread; however, its dense stromal microenvironment, enzymatic activity, and relative lack of sinusoidal vascular beds are thought to provide a less hospitable environment for metastatic seeding [9]. These physiologic and anatomic factors likely contribute to the infrequency of pancreatic metastasis across solid tumor types.

Although uLMS is a histologic diagnosis defined by established criteria, certain imaging features on MRI have been consistently described as concerning for uLMS. Prior radiologic studies have demonstrated that large uterine masses with marked heterogeneity, irregular margins, central necrosis, hemorrhage, and high signal intensity on T1‐ and T2‐weighted imaging are more frequently associated with uLMS than benign leiomyomas [10]. However, these imaging findings lack sufficient specificity to establish a definitive diagnosis, underscoring the limitation of radiography alone and the necessity of tissue confirmation. In the present case, imaging served to raise suspicion for uLMS and guide diagnostic prioritization, whereas histopathologic evaluation ultimately established the diagnosis.

In previously reported cases of uLMS with pancreatic involvement [11–16], pancreatic metastases were identified after hysterectomy for known uLMS and generally occurred as part of widespread systemic relapse rather than as an isolated synchronous lesion. Thus, this case differs from existing literature in two clinically significant ways: (1) The pancreatic lesion represented synchronous disease discovered at initial presentation rather than recurrent disease, and (2) the metastasis was isolated to the pancreas without concurrent pulmonary, hepatic, or peritoneal involvement. This distinction is important because it expands the known metastatic spectrum of uLMS and underscores that pancreatic involvement can occur in the absence of diffusely disseminated disease.

In this patient, the presence of a solitary pancreatic lesion alongside a large uterine mass prompted complex diagnostic considerations. The absence of more common metastatic sites, the relatively small size of the pancreatic lesion, and the lack of obstructive jaundice or constitutional symptoms made a primary pancreatic neoplasm a plausible alternative diagnosis.

The differential diagnosis for a solitary pancreatic lesion in this context is broad. Primary pancreatic adenocarcinoma typically presents with ductal dilation, jaundice, or constitutional symptoms, none of which were present. Pancreatic neuroendocrine tumors may present as small hypervascular lesions, whereas metastases from renal cell carcinoma, melanoma, or colorectal cancer can also occur. In our patient, synchronous colorectal malignancy was excluded through colonoscopy, and the absence of pulmonary or hepatic disease made widespread metastatic carcinoma less likely. However, without uterine tissue sampling, alternative diagnoses such as carcinosarcoma with sarcomatous overgrowth or dual primaries could not be definitely excluded. This limitation reflects a real‐world challenge in medically fragile patients, in whom invasive diagnostic procedures may not be feasible or may not alter management.

Pancreatic lesions can be sampled via CT‐ or IR‐guided percutaneous approaches or via EUS‐guided biopsy. CT‐guided biopsy is frequently used for peripheral pancreatic lesions due to accessibility, but pancreatic head lesions under 2–3 cm pose substantial challenges due to overlying bowel and proximity to major vessels. In contrast, EUS‐guided biopsy allows real‐time visualization and transduodenal access to deep pancreatic head lesions, making it the preferred diagnostic modality in many academic centers. However, EUS requires specialized training, equipment, and endoscopic expertise and may not be readily available in all institutions, particularly outside tertiary referral centers [8]. In this case, the lesion′s small size, central location, and vascular proximity made percutaneous biopsy higher risk, supporting the decision to pursue EUS‐guided tissue acquisition.

Therapeutic decision‐making in this case required integration of tumor biology, comorbidity burden, and patient goals. Metastasectomy or pancreaticoduodenectomy has been described for isolated pancreatic metastases from other solid tumors with suboptimal outcomes [13]. This with concurrent total abdominal hysterectomy could have certainly been a consideration in the appropriately selected surgical candidate, given that oncologic outcomes of leiomyosarcoma are improved with complete cytoreduction. Additionally, for unresectable oligometastatic lesions, SBRT may offer local control, though data in uLMS is limited. First‐line preferred systemic therapy options include doxorubicin with or without trametinib, docetaxel/gemcitabine, doxorubicin/ifosfamide, or dacarabazine/doxorubicin. These systemic therapy options are relatively toxic, each with a distinct set of side effects. In this case, doxorubicin with or without trabectedin was considered but not initiated due to the patient′s recent ischemic stroke, paroxysmal atrial flutter, and symptomatic anemia, which collectively conferred high risk for anthracycline‐associated cardiotoxicity, arrhythmia, and myelosuppression. Anthracyclines are known to cause dose‐dependent left ventricular dysfunction and precipitate arrhythmias [17], and thus require baseline cardiac reserve, which was not present in this patient. The LMS‐04 trial [18] demonstrated that trabectedin improves progression‐free survival following doxorubicin but carries significant hematologic toxicity, requiring careful patient selection. Given this patient′s poor performance status, cerebrovascular disease, and cardiac risk profile, these therapies were deemed unsafe and unlikely to provide meaningful benefit.

Ultimately, the patient elected hospice care. This decision underscores an essential but often underreported aspect of advanced sarcoma care: Prognosis and tolerability are dictated not only by tumor aggressiveness but also by comorbidity, functional status, and patient priorities. Incorporating palliative care early and emphasizing shared decision‐making are critical oncologic competencies, especially in rare presentations where evidence‐based guidance is limited.

## 4. Conclusion

Oligometastasis of uLMS to the pancreas is rare and diagnostically challenging. Recognition of atypical metastatic patterns and thoughtful multidisciplinary care are critical, particularly in patients for whom aggressive therapy may not be appropriate. This case contributes to the limited literature on pancreatic metastases from uLMS and underscores the critical role of definitive histopathologic diagnosis—supported by advanced diagnostic modalities—in guiding individualized, patient‐centered care.

## Funding

No funding was received for this manuscript.

## Consent

Written informed consent was obtained from the patient for publication of this case report and accompanying images.

## Conflicts of Interest

The authors declare no conflicts of interest.

## Data Availability

The data that support the findings of this study are available from the corresponding author upon reasonable request.
